# Effect of CO_2_ Enrichment on Synthesis of Some Primary and Secondary Metabolites in Ginger (*Zingiber officinale* Roscoe)

**DOI:** 10.3390/ijms12021101

**Published:** 2011-02-10

**Authors:** Ali Ghasemzadeh, Hawa Z. E. Jaafar

**Affiliations:** Department of Crop Science, Faculty of Agriculture, University Putra Malaysia, 43400 UPM Serdang, Selangor, Malaysia; E-Mail: upmali@yahoo.com

**Keywords:** CO_2_ enrichment, TSC, TF, TP, FRAP assay, Halia Bara

## Abstract

The effect of two different CO_2_ concentrations (400 and 800 μmol mol^−1^) on the photosynthesis rate, primary and secondary metabolite syntheses and the antioxidant activities of the leaves, stems and rhizomes of two *Zingiber officinale* varieties (Halia Bentong and Halia Bara) were assessed in an effort to compare and validate the medicinal potential of the subterranean part of the young ginger. High photosynthesis rate (10.05 μmol CO_2_ m^−2^s^−1^ in Halia Bara) and plant biomass (83.4 g in Halia Bentong) were observed at 800 μmol mol^−1^ CO_2_. Stomatal conductance decreased and water use efficiency increased with elevated CO_2_ concentration. Total flavonoids (TF), total phenolics (TP), total soluble carbohydrates (TSC), starch and plant biomass increased significantly (*P* ≤ 0.05) in all parts of the ginger varieties under elevated CO_2_ (800 μmol mol^−1^). The order of the TF and TP increment in the parts of the plant was rhizomes > stems > leaves. More specifically, Halia Bara had a greater increase of TF (2.05 mg/g dry weight) and TP (14.31 mg/g dry weight) compared to Halia Bentong (TF: 1.42 mg/g dry weight; TP: 9.11 mg/g dry weight) in average over the whole plant. Furthermore, plants with the highest rate of photosynthesis had the highest TSC and phenolics content. Significant differences between treatments and species were observed for TF and TP production. Correlation coefficient showed that TSC and TP content are positively correlated in both varieties. The antioxidant activity, as determined by the ferric reducing/antioxidant potential (FRAP) activity, increased in young ginger grown under elevated CO_2_. The FRAP values for the leaves, rhizomes and stems extracts of both varieties grown under two different CO_2_ concentrations (400 and 800 μmol mol^−1^) were significantly lower than those of vitamin C (3107.28 μmol Fe (II)/g) and α-tocopherol (953 μmol Fe (II)/g), but higher than that of BHT (74.31 μmol Fe (II)/g). These results indicate that the plant biomass, primary and secondary metabolite synthesis, and following that, antioxidant activities of Malaysian young ginger varieties can be enhanced through controlled environment (CE) and CO_2_ enrichment.

## 1. Introduction

The increase of CO_2_ concentration in the atmosphere is well documented. A stimulation of plant growth, photosynthesis rate and biochemical composition under elevated CO_2_ are shown in most of the recent reviews [1–3]. The response of plants to CO_2_ enrichment results in an increase in biomass accumulation, leaf area, or individual plant size [1,2]. Exposure of plants to elevated CO_2_ usually leads to increased rates of net photosynthesis due to enhanced activity of Rubisco enzyme and can alter plant growth and partitioning to secondary metabolites [2,3]. This can be proven from the result of the study by Wang: that elevated CO_2_ concentration in the atmosphere enhances vegetative growth, carbohydrate accumulation and fruit productivity in strawberry [4]. Leaves exposed to an enriched CO_2_ environment often show decreased diffusive conductance [5,6] and it is extensively assumed that elevated CO_2_ concentrations in the environment lead to reduced stomatal conductance [7]. Such a reduction of stomatal conductance and diffusion may result in reduced vapor losses per unit of CO_2_ assimilated [8] and usually translates into decreased rates of plant transpiration per unit leaf area and increased soil moisture in CO_2_-enriched conditions [9]. As carbon dioxide level doubled, stomatal conductance was shown to reduce by 30–40%, however there were variations among species [10]. Concomitant to this, water use efficiency (WUE) will also increase. This increase is caused more by increased net photosynthesis than by a reduction of water loss through partially closed stomata, thus, more dry matter can be produced per unit of water used [11]. Elevated atmospheric CO_2_ concentration often increases total non-structural carbohydrates (TNC) concentration in plants and possibly stimulates secondary metabolism [12]. While primary products, such as carbohydrates, lipids, proteins, chlorophyll, nucleic acids, *etc*., are involved in the primary metabolic processes of maintaining and building plant cells [13,14], secondary products of plants have historically been defined as chemicals that do not seem to have a critical biochemical role in such building and maintainince processes. Plants and herbs consumed by humans may contain thousands of different phenolic acid and flavonoid components. Currently, the effect of dietary phenolics is of great interest due to their antioxidative and possible anticarcinogenic activities [15]. Phenolic acids and flavonoids also function as reducing agents, free radical scavengers and quenchers of singlet oxygen formation [16–18]. Phenolic and flavonoid components have important roles to control cancer and diseases in human body [19,20]. There are also many reports provided that CO_2_ enrichment increases the production of secondary metabolites [2–4,21] and antioxidant activity of plants [3]. Increased concentration of flavonoids through CO_2_ enrichment has the potential to enhance the production and quality of medicinal plants such as Scutellaria. Increasing the phenolic and flavonoid components of *Populus tremuloides* by a CO_2_ enrichment method has been reported by Lindroth *et al.* [22]. According to the carbon-nutrient balance theory, as the carbon to nitrogen ratio increases under an elevated atmospheric CO_2_ environment, a greater amount of the plant’s carbohydrates can then be allocated to the plant’s secondary metabolism, resulting in the production of greater amounts of carbon based secondary metabolites [23]. Ginger is an important horticultural crop in tropical Southeast Asia. It is the most widely used herb especially in Asia and contains several interesting bioactive constituents and possesses health promoting properties [24]. Moreover, it can serve as a cheap and important material in food. Food composition and food additives play major role in providing the required antioxidants for the body, although, traditionally, spices such as ginger are commonly used in food preparations to improve the flavor and taste in Malaysia. Several researches have shown that spices containing phenolic and flavonoid compounds, showed antioxidant activities [25–28]. A positive linear correlation among phenolic compounds, flavonoids, and the antioxidant capacity of herbs and spices has also been established [14,29–31]. One imperative topic that has been ignored is the effect of elevated levels of atmospheric CO_2_ on the growth of medicinal plants and their production of secondary metabolites of therapeutic value. No information is available on the effect of CO_2_ concentration on the polyphenolic content and scavenging capacity against active oxygen species of Malaysian young ginger varieties. The objective of this study was to consider the effect of CO_2_ enrichment on biomass, leaf gas exchange, and primary and secondary metabolite synthesis in two varieties of Malaysian young ginger (*Zingiber officinale*), namely Halia Bentong and Halia Bara. The relationships among photosynthesis, carbohydrate, and total phenolics and flavonoids of plants exposed to CO_2_ enrichment were also determined.

## 2. Results and Discussion

### 2.1. Plant Biomass, Photosynthesis Rate, Water Use Efficiency

Several studies dealing with the influence of elevated CO_2_ levels on the growth and biochemical composition of plants have been conducted [32,33]. Dry weight of leaves, stems and rhizomes of ginger varieties were enhanced with rising CO_2_ (Table 1). With an increase in CO_2_ concentration from 400 to 800 μmol mol^−1^, total plant biomass was enhanced 47.6% in Halia Bentong and 76.3% in Halia Bara. The order of increase of biomass in both varieties under elevated CO_2_ concentration was rhizomes > leaves > stems. Buddendorf [34] suggested that the optimum CO_2_ concentration necessary to achieve the highest growth rates varies among species. Photosynthesis rate was increased in both of varieties under elevated CO_2_ concentration (Halia Bentong 65% and Halia Bara 46%). A stimulation of photosynthetic rate under elevated CO_2_ was shown in previous studies [35,36]. The increase carboxylation activity of ribulose 1,5-bisphosphate carboxylaseoxygenase enzyme (rubisco) in leaves under elevated carbon dioxide level increased net photosynthesis, especially in C3 species [36].CO_2_ enrichment decreased stomatal conductance significantly (*P* ≤ 0.05). Acclimation of stomata conductance under elevated CO_2_ may also be crucial to influence assimilation rates; a reduction of about 20% in plants was observed and the responses were variable depending on the environmental constraints, duration of the experiment and the species [37]. Low stomatal conductance and high WUE are often observed in plants grown in elevated CO_2_ concentrations [38]. Net photosynthesis was positively correlated with plant biomass production and negatively correlated with stomatal conductance (Table 2). This kind of correlation was reported by previous studies [1,2]. Elevated carbon treatments significantly increase WUE in both varieties (Table 1; *P* ≤ 0.05). This data shows that ginger needs little water to maintain turgidity of the plant cells when enriched with carbon dioxide. Water use efficiency was positively correlated with net photosynthesis and negatively related to stomatal conductance (Table 2). An increase in carbon dioxide concentration generally enhances photosynthesis and increases WUE [39]. Jackson *et al.* [40] defined water use efficiency as the amount of carbon dioxide assimilated through photosynthesis relative to the water lost through transpiration. Many researchers have ascribed this increase to greater net photosynthesis associated with greater carbon dioxide availability, lower transpiration resulting from lower stomatal conductance, or the combination of the two. Typically, WUE increased because stomata conductance and evapo-transpiration rates are reduced, whereas internal carbon dioxide level remain relatively constant [41].

### 2.2. Total Soluble Carbohydrate (TSC) and Starch Content

**Figure 1.**
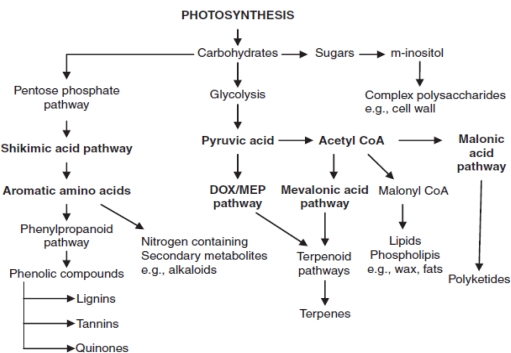
Principle biosynthetic pathway leading to synthesis of secondary metabolites [47].

It was found that elevated carbon dioxide concentration had significant effect on TSC and starch content. Maximum TSC content was observed in Halia Bara (38.43 mg/g dry weight) and Halia Bentong (38.22 mg/g dry weight) leaves grown under 800 μmol mol^−1^ CO_2_ (Table 3) and maximum starch content was observed in Halia Bentong rhizomes (583.5 mg/g dry weight) and Halia Bara rhizomes (553.3 mg/g dry weight) grown under 800 μmol mol^−1^ CO_2_ (Table 4). Elevated CO_2_ concentration enhanced TSC and starch content in all parts of both varieties. Due to elevated CO_2_, carbohydrates accumulate in plant tissues, as their usage intensity is lower than their production under these conditions [42,43]. Previous studies have reported that during growth under twice ambient CO_2_ level, the soluble carbohydrate content of leaves increased by 52% on average [44]. Plant cells produce two types of metabolites, primary and secondary. Primary metabolites are involved directly in the growth and metabolic processes, *viz.* carbohydrates, lipids and proteins. Additionally, they are produced as a result of photosynthesis and are also involved in cell component synthesis. Most natural products consist of compounds derived from primary metabolites such as amino acids, carbohydrates and fatty acids, and consequently, they are generally categorized as secondary metabolites (Figure 1). Carbohydrates are important compounds and have the most significant effects on flavonoids and phenolics production and partitioning in plant organs. Plant phenolics (flavonoid and phenols) are biosynthesized via several routes and thus constitute a heterogeneous group from the metabolic point of view. The two basic pathways involved are the shikimic acid and the malonic acid pathways. The shikimic acid pathway participates in the biosynthesis of most plant phenolics. Via this pathway, soluble carbohydrates are the basic component used to produce phenolic component. The shikimic acid pathway is able to convert simple carbohydrate precursors derived from glycolysis and pentose phosphate pathway to aromatic amino acids [45]. Previous studies have shown that the increase in phenolic concentration is related to the balance between carbohydrate sources and sinks, such that greater source or sink ratio results in higher phenolic concentration [45,46]. Based on the correlation Table 2, there was a significant relationship between TSC and TP. Enhancement of starch content by elevated CO_2_ concentration in rhizomes was greater than in the leaves of both varieties. It was found from the correlation (Table 2) that TSC and total phenolics and flavonoids were significantly (*P* ≤ 0.05) and positively related. On the other hand, elevated carbon dioxide enhanced soluble carbohydrate content, and in turn, enhanced phenolics and flavonoids synthesis in young ginger varieties.

### 2.3. Total Phenolic and Flavonoids

The content of flavonoids and phenolic components in methanolic extracts of the leaves, rhizomes and stems of the two varieties of *Z. officinale* are presented in Table 5. For both varieties grown under ambient (400 μmol mol^−1^) CO_2_, the total flavonoid and phenolic contents were highest in the leaves, followed by rhizomes then stems. When comparing the varieties when grown under ambient concentration of CO_2_, it was found that Halia Bara had higher TP (5.04 mg/g dry weight) and TF (1.27 mg/g dry weight) contents than Halia Bentong in the whole plant on average. The differences between the varieties and between the plant parts were highly significant (*P* ≤ 0.001). The total content of flavonoids and phenolics are influenced by the interaction between varieties and parts of plants. The CO_2_ enhancement resulted in significantly increased amounts of TP and TF in all parts of ginger varieties. Rhizomes in both varieties had higher increase of TF content at elevated CO_2_. According to the data in Table 5, TF content increased 1.42 mg/g dry weight in Halia Bentong and 2.05 mg/g dry weight in Halia Bara on average at elevated CO_2_. Between the different plant parts, TF content in Halia Bentong rhizomes increased by 3.32 mg/g dry weight, while Halia Bara increased by 5.3 mg/g dry weight. TP content also increased in ginger grown under 800 μmol mol^−1^ of CO_2_ (9.11 mg/g dry weight in Halia Bentong and 14.31 mg/g dry weight in Halia Bara). The increasing TP content for both varieties was higher in rhizomes (17.43 mg/g dry weight in Halia Bentong and 24.66 mg/g dry weight in Halia Bara). However, the order of increase of TF and TP in both of varieties was rhizomes > stems > leaves. Lavola *et al.* [31] reported that phenolic content increased in leaves and stems of *Betula pendula* grown under 700 μmol mol^−1^ CO_2_. He showed that certain flavonoid components such as catechin were detected from leaves of plants grown under elevated CO_2_. Stutte *et al.* [48] showed that increasing the CO_2_ concentration affected the concentration of flavonoids in the vegetative tissue of *S. barbata*, in which the combined concentration of the flavonoids measured increased by 48% at 1200 μmol mol^−1^ of CO_2_ concentration. Results of the present study showed that a increase in photosynthesis could have stimulated the production of plant secondary metabolites, as shown by the positive correlation coefficient (Table 2) between photosynthesis and total phenolics (*r*^2^ = 0.83). It was found from the correlation that photosynthesis and total flavonoids were significantly (*P* ≤ 0.05) and positively related. Nevertheless, the regression analysis exhibited a higher influence of soluble sugar concentration than starch on TP and TF biosynthesis.

### 2.4. Ferric Reducing/Antioxidant Potential (FRAP)

**Figure 2.**
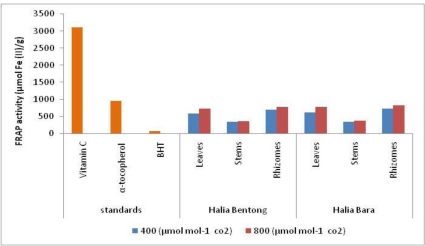
Effect of elevated CO_2_ on FRAP activity of the methanol extracts in different part of two varieties of *Z. officinale,* compared with positive controls: vitamin C, α-tocopherol and butylated hydroxytoluene (BHT).

Several methods are known to measure the total antioxidant capacity of herbs, including ferric reducing/antioxidant potential (FRAP) assay, which has been adopted in this study. The FRAP assay depends upon the reduction of ferric tripyridyltriazine (Fe (III)-TPTZ) complex to the ferrous tripyridyltriazine (Fe (II)-TPTZ) by a reductant at low pH. The reducing power for the different parts (leaves, stems and rhizomes) of young ginger extracts was in the range of 341.2–831.16 μm of Fe (II)/g dry weight (Table 6). Increasing CO_2_ concentration in the growth climate had significant effect on FRAP activities of young ginger parts. The FRAP values for the leaves, rhizomes and stems extracts in both varieties grown under two different CO_2_ concentrations (400 and 800 μmol mol^−1^) were significantly lower than those of vitamin C (3107.28 μmol Fe (II)/g) and α-tocopherol (953 μmol Fe (II)/g), but higher than that of BHT (74.31 μmol Fe (II)/g) (Figure 2). It was reported that the effect of antioxidant scavenging is due to hydrogen donating ability [29,49–52]. The FRAP assay has been used widely to estimate the antioxidant component/power in dietary polyphenols [52]. At ambient (400 μmol mol^−1^) and elevated CO_2_ (800 μmol mol^−1^), rhizomes of both varieties showed high reducing ability. The antioxidant potential (FRAP) of leaves and rhizomes of ginger varieties were greater than the stems at elevated CO_2_ concentration. It can be seen that CO_2_ enrichment significantly enhanced flavonol content in ginger varieties and further, high flavonol content was associated with high antioxidant activity. In a previous study, a strong positive relationship between total phenolic contents and antioxidant activity, which appears to be the trend in many plant species, was reported [53]. Significant correlation between FRAP acvtivity, TP and TF content was observed (Table 2). Wang *et al.* [4] reported that free radical scavenging power of strawberry increased at elevated CO_2_ concentration (950 μmol mol^−1^). This study has shown that ginger has good free radical scavenging ability and therefore can be used as a radical inhibitor or scavenger, acting possibly as a primary antioxidant. Additionally, increasing CO_2_ content in the environment can enhance the antioxidant activity of ginger extract, especially its rhizomes.

## 3. Experimental

### 3.1. Plants Material

Two varieties of *Zingiber officinale* Roscoe (Halia Bentong and Halia Bara) rhizomes were germinated for two weeks and then transferred to polyethylene bags filled with soilless mixture included burnt rice husk, coco peat with ratio 1:1. After two weeks, plants were transferred to a CO_2_ growth chamber (Conviron EF7, Canada) with two different CO_2_ concentrations, first, 400 μmol mol^−1^ as ambient and 800 μmol mol^−1^ as elevated CO_2_ concentration. Pure carbon dioxide (99.8 % purity; Company: ScienceGates Sdn Bhd) was supplied from a high concentration carbon dioxide cylinder (50 lbs, pressure 2200 PSI) and injected through a pressure regulator into the closed fumigation chamber. Photoperiod (310 μmol m^−2^s^−1^), relative humidity and air temperature of the chamber were controlled using integrated control, monitoring, and data management system software (Dynamac Corp., Rockville, MD, USA.). Plants were harvested after 16 weeks and leaves, stems and rhizomes were separated and after freeze drying kept at −80 °C until further analysis. The location of experiments was the Biosystem laboratory, the Faculty of Engineering, University Putra Malaysia (UPM).

### 3.2. Extract Preparation

Leaves, stems and rhizomes were dried (freeze dry) to constant weights. Leaves, stems and rhizomes (1 g) were powdered and extracted using methanol (50 mL), with continuous swirling for 1 h at room temperature using an orbital shaker. Extracts were filtered under suction and stored at −20 °C for further use.

### 3.3. Determination of Plant Biomass and Photosynthesis Rate

Plant harvesting was carried out at 16 weeks after planting. Nine plants of each CO_2_ level were chosen randomly and their total biomass was separated into three compartments: leaves, stems and rhizomes, and their dry weight were calculated after drying at 70 °C (72 h). Photosynthetic rate of fully expanded leaves was measured by using a portable photosynthesis system (LICOR-64001 LI-COR Inc., USA).

### 3.4. Determination of Total Soluble Carbohydrate (TSC)

A few drops of ethanol (80%) were added onto 0.1 g of freeze dried samples (leaves, stems, rhizomes). Then 25 mL of aqueous ethanol was added and mixed with shaking. Solutions were centrifuged at 5000 rpm. About 1 mL of supernatant was placed into test tubes and 10 mL of anthrone solution (0.15%) was added and finally the samples were heated. Tubes were cooled down to room temperature, then absorption of the samples was recorded at 625 nm [54].

### 3.5. Determination of Starch Content

Cold water (5 mL) was added to 6.5 mL of perchloric acid (52%) and mixed. Water (20 mL) was then added onto the residual material used for sugar analysis. The samples were centrifuged at 10,000 g. About 2.5 mL of supernatant were aliquoted into test tubes and 10 mL of cold anthron solution (2%) were added. The samples were then heated at 100 °C for 7.5 min. Tubes were cooled down to room temperature, then absorption of the samples was recorded at 630 nm [55].

### 3.6. Determination of Total Phenolic Content

The total phenolic content was determined following the method of Kim *et al*. [56]. Briefly, 1 mL of extract was added to deionized water (10 mL) and Folin–Ciocalteu phenol reagents (1.0 mL). After 5 min, 20% sodium carbonate (2.0 mL) was added to the mixture. The solution was kept in total darkness, and the absorbance was measured at 750 nm using a spectrophotometer (U-2001, Hitachi Instruments Inc., Tokyo, Japan).

### 3.7. Determination of Total Flavonoids

The TF were measured following a previously reported spectrophotometric method [57]. Briefly, extracts of each plant material (1 mL) were diluted with 4 mL water in a 10 mL volumetric flask. Initially, 5% NaNO_2_ solution (0.3 mL) was added to each volumetric flask; after 5 min, 10% AlCl_3_ (w/w) was added; and at 6 min, 1.0 M NaOH (2 mL) was added. Absorbance of the reaction mixture was read at 430 nm.

### 3.8. Determination of Antioxidant Activities

#### 

##### Reducing Ability (FRAP Assay)

The stock solutions included 300 mM acetate buffer, 10 mM TPTZ (2,4,6-tripyridyl-*s*-triazine) solution in 40 mM HCl, and 20 mM FeCl_3_ solution. Acetate buffer (25 mL) and TPTZ (2.5 mL) were mixed, and 2.5 mL FeCl_3_ added. Plant extracts (150 μL) were added to 2850 μL of the FRAP solution and kept for 30 min in the dark. The absorbance was measured at 593 nm using a spectrophotometer (U-2001, Hitachi Instruments Inc., Tokyo, Japan). [58].

### 3.9. Statistical Analysis

The experiments were split-split plot based on randomized complete block design (RCBD) and results were expressed as mean ± standard deviation. Where applicable, the data were subjected to one way analysis of variance (ANOVA) and the differences between samples were determined by Duncan’s Multiple Range test using the Statistical Analysis System (SAS, 1999) and MSTATC programs. *P* Values ≤ 0.05 were regarded as significant.

## 4. Conclusions

Ginger biomass and photosynthesis rate were enhanced when varieties were exposed to elevated CO_2_ concentration. Carbon dioxide enrichment increases net photosynthesis by increases availability of 3 phosphor-glycerate, 3PGA (triose phosphate), a carbohydrate precursor. Ginger varieties acclimatized by reducing transpiration and stomata conductance. Despite the reduction in transpiration and stomata conductance, water use efficiency of the plants increased. The increase in phenolics concentration under elevated CO_2_ was parallel to an increase in photosynthesis rate and to an increase in the TSC concentration, indicating higher availability of carbon to be invested in carbon based secondary compounds, which is also in accordance with source-sinks theories for carbon based secondary compounds [59]. It would appear that atmospheric CO_2_ enrichment not only significantly enhances biomass production in ginger varieties, but that it also slightly increases the concentrations of several therapeutic compounds. These results clearly demonstrate the potential of using controlled environment (CE) with elevated CO_2_ concentration to increase the primary metabolites and bioactive medicinal components such as flavonoids and phenolics in young ginger parts (leaf, stem, rhizome) especially in the leaves and rhizomes. Furthermore, positive and significant correlation was observed between TSC, TF, TP and FRAP activities. The results indicated that increasing atmospheric concentration of carbon dioxide could affect ginger’s antioxidant capacities significantly. It is important that this matter is properly understood, especially for the purpose of herb chemistry optimization.

## Figures and Tables

**Table 1.** Effect of different CO_2_ concentration on biomass, photosynthesis rate and stomata conductance of ginger varieties.

**Parameters**	**Halia Bentong**		**Halia Bara**	

	**400**	**800**	**400**	**800**
Leaves biomass	22.83 ±0.91^c^	35.3 ±0.46^b^	20.79 ±0.37^d^	38.5 ± 1.18a
Stems biomass	19.1 ±1.23^a^	23.8 ±0.47^a^	20 ±1.26^a^	24.4 ± 0.046^a^
Rhizomes biomass	14.5 ±0.29^b^	24.1 ±1.005^a^	6.91 ±0.049^c^	14.05 ± 0.61^b^
Total Biomass	56.5 ±1.85^c^	83.4 ±1.93^a^	47.7 ±0.84^d^	77.05 ± 1.76^b^
Photosynthesis	5.58 ±0.24^d^	9.22 ±0.35^b^	6.86 ±0.028^c^	10.05 ±0.14^a^
Stomatal conductance	0.182 ±0.005^a^	0.126 ±0.03^ab^	0.106 ±0.015^ab^	0.08 ± 0.009^b^
Water use efficiency	1.52 ±0.056^b^	1.85 ±0.035^a^	0.99 ±0.042^c^	1.48 ± 0.007^b^

Results of biomass expressed in g/plant; Results of Photosynthesis expressed in μmol CO_2_ m^−2^s^−1^; Results of Stomatal conductance expressed in mmol m^−2^s^−1^. All analyses are the mean ± standard deviation. Means not sharing a common letter were significantly different at *P* ≤ 0.05.

**Table 2.** Correlation between studied parameters.

		**1**	**2**	**3**	**4**	**5**	**6**	**7**	**8**	**9**
1	Photosynthesis	1								
2	Stomatal conductance	−0.56^n.s^	1							
3	WUE	0.87**	−0.81**	1						
4	Biomass	0.85**	−0.90**	0.86**	1					
5	TSC	0.96**	−0.72*	0.60^n.s^	0.93**	1				
6	Starch	0.92**	−0.74*	0.71*	0.92**	0.94**	1			
7	TP	0.83**	−0.24^n.s^	0.007^n.s^	0.49^n.s^	0.71*	0.72*	1		
8	TF	0.72*	−0.18^n.s^	0.07^n.s^	0.5^n.s^	0.70*	0.63^n.s^	0.9**	1	
9	FRAP	0.71*	−0.43^n.s^	0.22^n.s^	0.51^n.s^	0.72*	0.49^n.s^	0.76*	0.71*	1

*, significant at *P* ≤ 0.05;

**, significant at *P* ≤ 0.01; n.s, non significant.

TF, total flavonoids; TP, total phenolics; WUE, water use efficiency.

**Table 3.** Total soluble carbohydrate content in different parts of ginger (*Z. officinale*) varieties grown under different CO_2_ concentration.

**Varieties**	**Parts**	**CO_2_ Concentration (μmol mol^−1^)**	
		**400**	**800**
Halia Bentong	Leaves	14.05 ± 0.953^e^	38.22 ± 1.98^a^
	Stems	11.8 ± 0.455^f^	17.26 ± 0.385^d^
	Rhizomes	10.9 ± 0.481^f^	27.63 ± 1.69^c^

Halia Bara	Leaves	14.46 ± 0.98^e^	38.43 ± 0.935^a^
	Stems	11.59 ± 1.8^f^	18.83 ± 0.895^d^
	Rhizomes	11.46 ± 0.63^f^	30.16 ± 2.004^b^

Expressed in units of mg/g dry weight; All analyses are the mean ± standard deviation; Means not sharing a common letter were significantly different at *P* ≤ 0.05.

**Table 4.** Starch content in different parts of ginger (*Z. officinale*) varieties grown under different CO_2_ concentration.

**Varieties**	**Parts**	**CO_2_ Concentration (μmol mol^−1^)**	
		**400**	**800**
Halia Bentong	Leaves	311.67 ± 14.4^c^	385.4 ± 10.9^b^
	Rhizome	311.9 ± 28.4^c^	583.5 ± 24.9^a^

Halia Bara	Leaves	317.2 ± 8.2^c^	402.7 ± 15.6^b^
	Rhizome	315.9 ± 15.2^c^	553.3 ± 24.6^a^

Expressed in units of mg/g dry weight; All analyses are the mean ± standard deviation; Means not sharing a common letter were significantly different at *P* ≤ 0.05.

**Table 5.** Total phenolic and flavonoid contents of the methanol extracts in different parts of two varieties of *Z. officinale.*

**Varieties**	**Plant Parts**	**TF (mg Quercetin/g dry weight)**		**TP (mg Gallic acid/g dry weight)**	
		**CO_2_ Concentration (μmol mol^−1^)**		**CO_2_ Concentration (μmol mol^−1^)**	

		**400**	**800**	**400**	**800**
Halia Bentong	Leaves	5.44 ±0.45^de^	6.04 ±0.79^d^	31.22 ±2.41^d^	39.68 ±5.61^c^
	Stems	1.61 ±0.22^g^	1.96 ±0.17^g^	6.14 ±0.8^f^	7.6 ± 0.66^ef^
	Rhizomes	4.03 ±0.081^f^	7.35 ±1.99^c^	11.33 ±0.27^e^	28.76 ±7.74^d^

Halia Bara	Leaves	8.66 ±0.42^bc^	9.23 ±0.36^ab^	43.22 ±2.15^b^	60.69 ±2.6^a^
	Stems	1.74 ±0.37^g^	2.04 ±0.31^g^	7.1 ±1.04^ef^	7.89 ± 1.17^ef^
	Rhizomes	4.48 ±0.08^ef^	9.78 ±0.77^a^	13.5 ±0.26^e^	38.16 ± 1.55^c^

TF and TP are total flavonoids and total phenolics contents; All analyses are the mean ± standard deviation. Means not sharing a common letter were significantly different between species and treatments at *P* ≤ 0.05.

**Table 6.** Total antioxidant (FRAP) activity in different part of two varieties of *Z. officinale*.

**Varieties**	**Extraction source**	**CO_2_ Concentration (μmol mol^−1^)**	
		**400**	**800**
Halia Bentong	Leaves	577.21 ± 14.21^e^	722.31 ± 11.45^d^
	Stems	341.2 ± 40.76^f^	356.31 ± 41.6^f^
	Rhizomes	671.3 ± 21.2^d^	774.62 ± 26.35^bc^

Halia Bara	Leaves	620.1 ± 18.7^e^	783.09 ± 21.95^b^
	Stems	350.2 ± 27.6^f^	367.4 ± 20.43^f^
	Rhizomes	740.6 ± 40.15^cd^	831.16 ± 39.08^a^

Expressed in units of μmol Fe (II)/g. All analyses are the mean of triplicate measurements ± standard deviation. Means not sharing a common letter were significantly different at *P* ≤ 0.05.
